# Methylprednisolone in Pediatric Cardiac Surgery: Is There Enough Evidence?

**DOI:** 10.3389/fcvm.2021.730157

**Published:** 2021-09-22

**Authors:** Annewil van Saet, Gerdien A. Zeilmaker-Roest, Robert J. Stolker, Ad J. J. C. Bogers, Dick Tibboel

**Affiliations:** ^1^Department of Anesthesiology, Erasmus Medical Center, Rotterdam, Netherlands; ^2^Department of Intensive Care and Pediatric Surgery, Erasmus Medical Center, Rotterdam, Netherlands; ^3^Department of Cardiothoracic Surgery, Erasmus Medical Center, Rotterdam, Netherlands

**Keywords:** methylprednisolone, cardiac surgery, pediatric, corticosteroids, pharmacokinetics

## Abstract

Corticosteroids have been used to decrease the inflammatory response to cardiac surgery and cardiopulmonary bypass in children for decades. Sparse information is present concerning the pharmacokinetics and pharmacodynamics of corticosteroids in the context of pediatric cardiac surgery. There is large interindividual variability in plasma concentrations, with indications for a larger volume of distribution in neonates compared to other age groups. There is ample evidence that perioperative use of MP leads to a decrease in pro-inflammatory mediators and an increase in anti-inflammatory mediators, with no difference in effect between doses of 2 and 30 mg/kg. No differences in inflammatory mediators have been shown between different times of administration relative to the start of surgery in various studies. MP has been shown to have a beneficial effect in certain subgroups of patients but is also associated with side effects. In lower risk categories, the balance between risk and benefit may be shifted toward risk. There is limited information on short- to medium-term outcome (mortality, low cardiac output syndrome, duration of mechanical ventilation, length of stay in the intensive care unit or the hospital), mostly from underpowered studies. No information on long-term outcome, such as neurodevelopmental outcome, is available. MP may provide a small benefit that is easily abolished by patient characteristics, surgical techniques, and perfusion management. The lack of evidence leads to large differences in practice between and within countries, and even within hospitals, so there is a need for adequately powered randomized studies.

## Introduction

The use of corticosteroids to decrease the inflammatory reaction to cardiac surgery with cardiopulmonary bypass (CPB) has become commonplace, even though there are limited convincing data of a beneficial impact on outcome (1). Since few articles have been published describing the actual corticosteroid plasma concentrations achieved, it is unknown whether this lack of a beneficial impact on outcome is caused by insufficient plasma concentrations reached before, during, or after CPB.

Research in adult patients cannot be extrapolated to children as the inflammatory response to CPB is different from adults (2–7).

In this review, we will focus on methylprednisolone (MP) and the pediatric population (Table 1). With a randomized double-blind placebo-controlled study currently underway (28) we decided to review what is known, what is not known, and how the Steroids to Reduce Systemic Inflammation After Infant Heart Surgery (STRESS) trial (https://clinicaltrials.gov/ct2/show/NCT03229538) may help us along in our quest for an answer to the question: who exactly are we treating when we give MP in pediatric cardiac surgery?

**Table 1:** Summary of methylprednisolone studies.

**Authors**	**Methods**	**Study group**	**Laboratory results**	**Clinical results**	**Side effects**
Pesonen et al. (8) Single-center RCT	MP 30 mg/kg at induction of anesthesia, MP 30 mg/kg in the CPB prime or placebo	45 children VSD or AVSD closure, mean age 5.3 months, mean weight 5.7 kg	Heart-type fatty acid-binding protein increased in all study groups with a maximum value after administration of protamine Heart-type fatty acid-binding protein lower at 6 h postoperative in both MP groups: induction group p = 0.01, prime group p = 0.03. Highly significant correlation of heart-type fatty acid-binding protein with TnT at protamine administration and 6 h postoperative.	Inotrope score at arrival in the ICU ns Inotrope score on POD1 ns	
Abbasi Tashnizi et al. (9) Single-center RCT	MP 30 mg/kg 4 h before CPB and in the priming fluid vs. MP 30 mg/kg 4 h before CPB	50 children Mean age 3.5 years	IL-6 ns IL-10 ns		
Pasquali et al. (10) Database study of the Pediatric Health Information Systems containing data from 39 participating US centers	54% of patients received steroids: MP 69.8%, dexamethasone 26.6%, other 3.6% Timing day of surgery only 79.1%, day before and day of surgery 12.2%, day before surgery only 8.9% Steroid use associated with younger age, prematurity, genetic syndrome, high RACHS-1 category, and south or north central US	46,730 children, median age 8 months Neonates < /= 30 days 21.7% Infants 34.5%		Mortality ns Duration of mechanical ventilation ns No significant benefit of corticosteroids in any RACHS-1 risk category	LOS ICU longer in corticosteroid patients overall and neonates p < 0.001 LOS hospital longer in corticosteroid patients p < 0.001 Infections more in corticosteroid patients p = 0.001 Use of insulin higher in corticosteroid patients overall and neonates p < 0.001 Morbidities associated with RACHS-1 1–3 category patients
Graham et al. (11) Multicenter RCT	MP 30 mg/kg after induction of anesthesia vs. placebo	176 neonates </= 30 days, mean age 8.7 days, mean weight 3.3 kg	Peak glucose ns	Primary endpoint (composite death, cardiac arrest, ECMO, renal injury, hepatic injury, elevated lactate) ns overall, protective in palliative surgery (OR 0.38, CI 0.15–0.99, p = 0.048) but not corrective surgery (OR 1.06, CI 0.45–2,47) Individual components primary outcome ns Duration of mechanical ventilation ns LOS ICU ns LOS hospital ns Fluid balance ns AKI ns LCOS ns	
				Inotrope score lower at 4 and 8 h postop in MP group p = 0.03 Lower highest inotrope-score first 36 h postoperatively Insulin treatment ns Infection ns Poor wound healing ns No difference in primary endpoint in children <7 days vs. children > 7 days. Interaction of center in which surgery takes place and treatment effect of MP; OR for primary endpoint overall center 1 0.35 (CI 0.15–0.84, p 0.02), center 2 OR 5.13 (0.85–30.9) though a protective effect in palliative surgery. In center 1 also reduced incidence of LCOS and inotrope score.	
Pasquali et al. (12) Database study of the STS-Congenital Heart Surgery database linked to data from Pediatric Health Information Systems containing data from 30 participating US centers	38% received no MP, 22% received MP on the preoperative and operative day, 28% on the operative day only, 12% on the preoperative day only.	3180 neonates </= 30 days from 25 US centers; 6 days old, 3.2 kg		LOS ICU ns LOS hospital ns	In-hospital mortality ns Infection ns
Keski et al. (13) Single center RCT	MP 30 mg/kg vs. placebo after induction of anesthesia	40 neonates mean age 10.5 days, mean weight 3.6 kg	Total MP peak concentration 4,000 ng/ml, unbound MP peak concentration 1,800 ng/ml IL-6 significantly lower 4 h after MP IL-8 significantly lower 4 h after MP IL-10 significantly higher 4 h after MP WBC on arrival at ICU higher in MP group p < 0.001 on POD 1 TnT ns SvO2 ns No correlation between total and unbound MP concentrations and cytokine levels or TnT	Inotrope score ns Mechanical ventilation days ns LOS ICU ns Mortality 30 days ns Wound infection ns	Lactate on arrival at ICU borderline higher in MP group p = 0.054 Glucose on arrival at ICU and POD 1 significantly higher in the MP group Significantly more patients received insulin in the MP group
Suominen et al. (14) Single center RCT	MP 2 mg/kg after anesthesia induction followed by hydrocortisone 0.2 mg/kg/h starting 6 h after weaning from CPB continued for 48 h, followed by 0.1 mg/kg/h for 48 h and then 0.05 mg/kg/h for 24 h vs. placebo	40 neonates Mean age 8.1 days, mean weight 3.5 kg	MP peak concentration 150 ng/dL IL-6 lower in MP group 6 h after weaning from CPB and POD1 and 2 p < 0.001 IL-10 higher 5 min after initiation of CPB in MP group p < 0.001 CRP lower at POD1-5 in MP group p < 0.001 WBC higher at POD1-4 in MP group p < 0.001 SvO2 ns Preoperative cortisol ns Postoperative cortisol ns ACTH stimulation test no suppression of hypothalamic–pituitary–adrenal axis ns Lactate 6 h after weaning from CPB higher in the MP group p < 0.001	Duration of mechanical ventilation ns Lower systemic ventricle delta strain values at 4 days in MP group p = 0.01, but not at 2 weeks p = 0.28 LVEF ns Inotrope score lower on POD1, 3 and 6 in the MP group p = 0.05 Sternal closure 2 days earlier in MP group p = 0.03 LOS ICU ns Death ns Postoperative infection ns Arrhythmias ns Fluid balance ns	Hyperglycemia 6 h after weaning from CPB and POD1 more prevalent in MP group p = 0.002 without a difference in insulin administration
Keski et al. (15) RCT single center	MP 30 mg/kg at induction of anesthesia, MP 30 mg/kg in the CPB prime or placebo	45 children for VSD or AVSD repair Mean age 5.3 months, mean weight 5.7 kg	Total MP peak concentration 7,000 ng/ml IL-6 ns IL-8 after weaning from CPB and 6 h postoperative decreased in induction group vs. prime (p = 0.003, p = 0.007) and placebo (p = 0.002, p = 0.001) IL-10 ns TnT 6 h postoperative significantly higher in the placebo group vs. induction group (p = 0.001) and prime group (p = 0.002). Lactate ns SvO2 ns WBC on POD 1 higher in treatment groups (p = 0.02)	Inotrope score ns Duration of mechanical ventilation ns LOS ICU ns	Hyperglycemia Leukocytosis
Keski et al. (16) RCT single-center	MP 30 mg/kg vs. MP 5 mg/kg at anesthesia induction	30 children median age 5.6 months, median weight 7 kg Primary TOF repair	Total MP peak concentrations after 30 mg/kg 8 500 ng/ml, after 5 mg/kg 1 000 ng/ml IL-6 ns IL-8 ns IL-10 ns Leukocytes ns TnT ns Lactate ns SvO2 ns NT-proBNP ns Higher blood glucose measurements after 30 mg/kg	30-day follow up Deaths none Duration of mechanical ventilation ns Inotropic score ns	Higher rates of administration of insulin after 30 mg/kg Two infections after 30 mg/kg Borderline more JET or AT after 30 mg/kg (p 0.06)
Grosek et al. (17) Prospective observational study	MP 30 mg/kg in CPB-prime Cardiac index <3 l/min/m^2^ defined as low cardiac index, cardiac index > 3 l/min/m^2^ defined as normal cardiac index	10 children for VSD repair 18.7 months, 9.3 kg	MP succinate concentrations immediately after CPB 13 200 ng/ml after 24 h 4,560 ng/ml MP concentrations immediately after CPB 7,400 ng/ml, after 24 h 34.9 ng/ml No differences in serum concentration of MP succinate, MP, or cortisol between groups. No difference in exposure to MP (AUC) between cardiac index groups Pharmacokinetics: MP succinate clearance 5 l/h, volume of distribution 1.5 l/kg MP clearance 4.13 l/h, volume of distribution 1 l/kg No significant influence of cardiac index on model parameters Normal cardiac index group lower HLA-DR+ monocytes immediately after CPB, no difference at 24 h. Normal cardiac index group lower T-lymphocytes, T_helper_ and T_cytotoxic_ lymphocytes at 24 h. Monocytes and cytotoxic T cells were lower in all groups immediately after CPB and remained lower at 24 h. T cells and T_helper_ cells were lower in all groups at 24 h.	Duration of mechanical ventilation ns LOS ICU ns Fluid balance ns	
Bocsi et al. (18) Retrospective analysis	10 patients received no MP, 23 received MP 11 mg/kg before CPB	33 children 5.2–9.7 years old, 15–27 kg for ASD or VSD repair	Total MP plasma concentration 11,900 ng/l	Duration of mechanical ventilation ns LOS ICU ns LOS hospital ns Chest tube output ns	
Hornik et al. (19) Population PK/pharmacodynamic model	MP 30 mg/kg vs. MP 10 mg/kg MP single dose *via* CPB prime vs. dose *via* CPB prime and preoperative dose	64 neonates < /= 30 days	MP plasma concentrations after a dose of 30 mg/kg ranged from 1000 to 7500 ng/ml. Clearance was calculated as 3.88 l/h and volume of distribution of the central compartment as 8.9 l No significant difference in concentration time profiles of IL-6 and IL-10 between 30- and 10-mg/kg dose groups and between single dose and addition of a second dose of MP 10 h before surgery. Similar results are obtained when stratification by PMA, RACHS-1 category, or CPB time takes place.		
Varan et al. (20) RCT single-center	MP 30 mg/kg vs. MP 2 mg/kg before the onset of CPB	30 children mean age 4 years, mean weight 13.7 kg	IL-6 ns IL-8 ns CRP ns Leucocytes ns Neutrophils ns	Duration of mechanical ventilation ns LOS ICU ns Blood loss ns Diuresis ns Blood urea nitrogen and creatinine ns ASAT/ALAT ns Cumulative inotropic score ns PaO_2_/FiO_2_ ratio ns	Infection ns Other nonspecified ns
Graham et al. (21), (22) RCT single-center	MP 30 mg/kg two dose 8 h preoperative and intraoperative in the CPB prime vs. one dose intraoperative	76 neonates mean age 8.2 days, mean weight 3.2 kg Corrective and palliative procedures	WBC higher immediately postoperative in two-dose group p = 0.04 40% lower IL-6 and TNF-α, 5-fold higher IL-10 at incision in the two-dose group Two-fold increase in IL-6 postoperatively ns IL-8 absolute values ns, when adjusted for baseline p < 0.01 at incision favoring two doses	LCOS ns Inotrope score ns Diuresis ns Duration of mechanical ventilation ns LOS ICU ns LOS hospital ns Higher baseline levels of IL-6, IL-8, IL-10, and TNFα associated with longer LOS ICU and LOS hospital	At 30 days 1 patient deceased in the one-dose group due to sepsis Plasma creatinine higher in the first 36 h in the two-dose group p = 0.03
			IL-6 after MUF ns IL-8 postoperative ns IL-10 after MUF higher in the two-dose group p < 0.01, ns at other postoperative time points IL-2 ns INF-γ ns	Postoperative response of IL-8 associated with LOS ICU and LOS hospital	Trend toward higher lactate (p = 0.05) and worse fluid balance (p = 0.052) in the first 36 h in the two-dose group
Withington et al. (23) RCT single center	MP 30 mg/kg evening before surgery vs. 30 mg/kg at anesthesia induction vs. 30 mg/kg in the CPB prime	54 children mean age 1.5 months, mean weight 4 kg	IL-6 baseline higher, with a higher rise in preoperative day group IL-8 ns Less oxidative stress in CPB prime, delayed increase in the preoperative group Preoperative dose group generally had more evidence of inflammation and oxidative stress.	Duration of mechanical ventilation ns LOS ICU ns but trend toward lower score in the pump prime group Inotrope score ns, but trend toward a lower score in the pump prime group A-aDO2 at 72 h ns Fluid balance ns Diuresis ns	
Crawford et al. (24) Retrospective analysis	MP 10 mg/kg 8 and 1 h before surgery followed by hydrocortisone or placebo 1.5 h after CPB	40 neonates for complex cardiac surgery	TNF-α, IL-6, and IL-10 were increased post CPB compared to preoperative		Incidence of adrenal insufficiency 32.5% with increased incidence of LCOS after MP administration perioperative if stress dose hydrocortisone is not administered.
Gibbison et al. (25) Systematic review of 8 RCT's	All types of corticosteroids, all dosing schemes, administered *via* any route and at any time-point in the perioperative period	478 children from birth up to 18 years of age undergoing cardiac surgery with CPB receiving prophylactic corticosteroids		In-hospital mortality ns All-cause mortality at longest follow-up ns Cardiovascular mortality at longest follow-up ns Duration of mechanical ventilation was decreased LOS ICU ns LOS hospital ns	
Li et al. (26) Systematic review and meta-analysis of 17 RCTs		84 pediatric patients undergoing cardiac surgery with CPB	Serum lactate on POD 1 ns	All-cause in-hospital mortality ns Inotrope score lower at POD 1 (p = 0.04) LOS ICU ns Duration of mechanical ventilation ns LCOS incidence ns Diuresis ns AKI ns Maximum temperature POD 1 ns	Hyperglycemia incidence increased on POD 1 (p = 0.0001) Risk of insulin therapy 2.69-fold higher (p = 0.004) Infection ns
Elhoff et al. (27) Retrospective multicenter database study	Neonates for Norwood from a randomized study Sano vs. MBT shunt Perioperative corticosteroid as dichotomous value	498 neonates received corticosteroids, 51 did not		Trend toward increased mortality in patients treated with corticosteroids in multivariate analysis. Neurodevelopmental outcome ns LOS ICU ns LOS hospital ns Need for cardiopulmonary resuscitation ns, trend toward improvement in patients not receiving corticosteroids Need for ECMO ns, trend toward improvement in patients not receiving corticosteroids AKI ns Infection ns	

*ACTH, adrenocorticotropin hormone; AKI, acute kidney injury; ASD, atrial septal defect; AT, atrial tachycardia; AVSD, atrioventricular septal defect; AUC, area under the curve; CI, confidence interval; CPB, cardiopulmonary bypass; CRP, Creactive protein; ECMO, extracorporeal membrane oxygenation; HLA-DR, human leukocyte antigen -DR isotype; ICU, intensive care unit; IFN, interferon; IL, interleukin; JET, junctional ectopic tachycardia; LCOS, low cardiac output syndrome; LOS, length of stay; MBT, modified Blalock -Taussig shunt; M, methylprednisolone; MUF, modified ultrafiltration; ns, not significant; NT-proBNP, N-terminal brain natriuretic peptide; OR, odds ratio; POD, postoperative day; RACHS-1 Risk, Adjustment in Congenital Heart Surgery; RCT, randomized controlled trial; STS, Society of Thoracic Surgeons; TNF, tumor necrosis factor; TnT, troponin T; TOF, tetralogy of Fallot; VSD, ventricular septal defect; US, United States of America; WBC, white blood cells*.

## Corticosteroid Mechanism of Action

Corticosteroids are the synthetic analogs of glucocorticoids. Glucocorticoids exert their actions by binding to glucocorticoid receptors (GR) that are localized in the cytoplasm of virtually all cell types and tissues. Binding can cause non-genomic, rapid onset effects by directly modulating signal transduction pathways *via* membrane-bound GR or cytosolic GR interactions with kinases (29).

Delayed effects of corticosteroids are mediated through pharmacogenomic pathways (29). The primary anti-inflammatory mechanism of action of corticosteroids is to repress pro-inflammatory genes encoding cytokines, chemokines, cell adhesion molecules, inflammatory enzymes, and receptors. Upon binding of hormone to the GR receptor, the receptor–hormone complex translocates to the cell nucleus. The transcription of target genes is repressed by direct binding to specific DNA sequences, tethering to transcription factors such as nuclear factor-κB (NF-κB) and activator protein (AP)-1 inhibiting their actions or direct binding to DNA and interacting with neighboring transcription factors (29).

More recently, mechanisms of corticosteroid action have also been shown to involve transactivation. A key protein in the mediation of glucocorticoid anti-inflammatory and immunosuppressive function is glucocorticoid-induced leucine zipper (GILZ or *Tsc22D3-2*) (30). GILZ is located on the X chromosome, and its expression is rapidly regulated by glucocorticoids. GILZ shows a variety of protein interactions, including inhibition of cyclo-oxygenase-2, NF-κB, and AP-1 (30). It is abundant in several cell types (30). In macrophages and monocytes, GILZ decreases pro-inflammatory cytokine and chemokine secretion. It prevents effective antigen presentation and the induction of regulatory T cells by dendritic cells and negatively regulates T-cell function, causing immunosuppression.

Genomic mechanisms of action take time to take effect. The nuclear translocation of a glucocorticoid receptor takes 30–60 min and changes in protein synthesis several hours (8). The peak effect of MP occurs 1–4 h after administration with a duration of action of 12–24 h (1, 9).

Glucocorticoids also regulate the immune response at the cellular level. They can induce apoptosis of T-cells and neutrophils to reduce inflammation, modulate the activation of both Th1 and Th2 cells, and regulate T-cell and macrophage expression of pro-inflammatory molecules (29).

Aside from transcriptional mechanisms, GR can also regulate the inflammatory response through a posttranscriptional mechanism by induction of the expression of proteins that block pro-inflammatory pathways (29).

## Corticosteroids in Pediatric Cardiac Surgery Patients

### Incidence of Use

Corticosteroids are commonly used during pediatric cardiac surgery with CPB. A survey in the United States and 14 other countries performed in 2005 showed that 96% of responding centers administered corticosteroids, although only 40% used corticosteroids in every case (1). Administration of corticosteroids is highly variable in the US. Fifty-four percent of patients in a database study received corticosteroids, with a range of 1–96% incidence of use between individual centers (10). There was no correlation between center volume and corticosteroid use. Corticosteroids were used most often in the southern and northern central regions of the US (10). In the US, corticosteroids are momentarily used in 51.2% of infant cardiac surgeries according to the Society of Thoracic Surgeons (STS) Congenital Heart Surgery Database, which collects anesthesia data since 2014 (28).

In the United Kingdom and Ireland, corticosteroids were used in 80% of pediatric cardiac centers in 2009, with 33% of these centers using them in every case (31). In a more recent survey from the United Kingdom, 35.1% of respondents reported not using any corticosteroids at all (32). 29.1% of anesthesiologists who use corticosteroids give them in all their cases (32). In the UK, there is also variability between and even within centers when it concerns corticosteroid treatment (32).

The variation in incidence of use between countries, within countries, and even within hospitals highlights the lack of evidence regarding the use of corticosteroids in pediatric cardiac surgery.

### Indications

In different surveys, different indications are mentioned for the use of corticosteroids. The most commonly named indications are surgery in neonates (1, 31, 32) and use of deep hypothermic circulatory arrest (DHCA)(1, 31, 32). Other indications named are surgeon preference, other operative characteristics such as expected long CPB time, Norwood operation or reoperation, higher Risk Adjustment in Congenital Heart Surgery (RACHS-1) (33) category, prematurity, and genetic abnormalities (1, 31, 32).

A randomized study of 174 neonates undergoing cardiac surgery was performed, comparing MP after induction of anesthesia vs. placebo. No influence of MP on a composite primary outcome of death, cardiac arrest, extracorporeal membrane oxygenation (ECMO), acute kidney injury (AKI), hepatic injury, or lactate > 5 mmol/l was shown (11). There was no difference in the effect of MP in neonates younger or older than 7 days. The study did, however, show a beneficial effect in a prespecified subgroup analysis involving neonates undergoing palliative procedures (OR 0.38, 95% confidence interval (CI) 0.15–0.99, p 0.048) (11).

An overall lack of effect of MP in neonates has also been shown in a large database study in 3,180 neonates in 25 US centers. In another database study of the same group, a subgroup of 10,018 neonates from 38 US centers had a longer length of stay (LOS) in the intensive care (ICU) and a higher use of insulin associated with corticosteroid use (10).

Morbidities associated with corticosteroid use are more prevalent in low-risk surgical patients. In a database study in 46,730 pediatric patients from 38 US centers, patients in risk category RACHS-1 1–3 showed a stronger association between corticosteroid use and morbidities (10). This result was confirmed in another database study of 3,180 neonates, which showed that in the group of patients with STS-European Association of Cardiothoracic Surgeons (34) risk categories 1–3, there was an association between the use of any MP regimen and infection (OR 2.6, 95% CI 1.3–5.2) (12). It is thus likely that in lower-risk categories the balance between risk and benefit is shifted toward risk. Unfortunately, in both studies it was not possible to evaluate the effect of different dosing regimens or timing of MP in relation to surgery on the occurrence of side effects. It is thus not known whether side effects can be prevented or whether their impact can be decreased by using a lower-dose or a single-dose regimen.

In a randomized controlled study comparing MP 30 mg/kg to placebo in neonates, MP was protective for the primary composite endpoint (death, cardiac arrest, ECMO, AKI, hepatic injury, elevated lactate) for palliative surgery, but not for corrective surgery (11). The center in which surgery took place interacted with the treatment effect of MP (11). MP was protective for the primary composite endpoint overall and at 30 days in one of two centers. The incidence of low cardiac output syndrome (LCOS) and inotrope requirement were also lower in patients treated with MP. In the other center, there was a lack of effect, and even a trend in the opposite direction. The authors hypothesized that MP may provide a small benefit that is easily abolished by patient characteristics or perioperative management strategies that are larger contributors to poor outcome.

### MP Exposure

Before conclusions can be drawn about the efficacy of a drug, it is important to ascertain that a sufficient concentration of the drug is reached in the plasma or even the effect site after administration.

In a group of 40 neonates receiving 30 mg/kg of MP after the induction of anesthesia, total and unbound MP plasma concentrations were 4,000 ng/ml and 1,900 ng/ml 30 min after the initiation of CPB (13). Even though these high concentrations induced lower concentrations of pro-inflammatory and higher concentrations of anti-inflammatory mediators, no differences in outcome parameters such as troponin-T (TnT), central venous oxygen saturation (SvO2), inotrope score, duration of mechanical ventilation, LOS in the ICU, or 30-day mortality were shown. In a group of 40 neonates receiving MP 2 mg/kg, the same research group has reported total MP concentrations of 150 ng/ml (14).

In a group of infants undergoing ventricular septum defect (VSD)—or atrioventricular septum defect (AVSD)—repair receiving 30 mg/kg of MP after the induction of anesthesia or in the priming fluid of the CPB system, concentrations of MP were 7,500 and 6,000 ng/ml after 30 min on CPB, suggesting no breakdown of MP in the prime fluid (15). In another study in infants for tetralogy of Fallot (TOF) repair by the same research group, MP concentrations 30 min after the initiation of CPB were 8,500 ng/ml after a dose of 30 mg/kg and 1,000 ng/ml after a dose of 5 mg/kg (16). Even with this marked difference in concentrations, no differences were shown in the concentrations of pro- and anti-inflammatory cytokines or in clinical outcome measures such as TnT, SvO2, inotrope score, N-terminal pro-B-type natriuretic peptide (NT-proBNP), duration of mechanical ventilation (DMV), and LOS in the ICU. The authors noted that total MP concentrations were twice as high compared to the total MP concentrations in neonates. They also noted marked interindividual variability in plasma concentrations and suggested that the use of population pharmacokinetic (PK) analysis may be needed for dose optimization of MP in pediatric cardiac surgery.

The high interindividual variability in MP concentrations after a dose of 30 mg/kg in the priming fluid of the CPB system was also shown in an earlier observational study of 10 infants undergoing VSD repair (17). Even though MP is a high extraction drug, lower cardiac index (CI) was not associated with decreased drug elimination, and thus increased drug exposure in this study. An exploratory population PK analysis showed similar PK parameters in low and normal CI children (17). Clearance was calculated as 4.13 l/h and volume of distribution (Vd) as 1.01 l/kg for infants with a body weight of 10 kg. In another study including older children (5.2–9.7 years old, 15–27 kg), an MP half-life of 2.62 h (median; interquartile range 2.53–2.93) was calculated after a dose of 11 mg/kg (18).

A population PK/pharmacodynamics model has since been published for neonates undergoing cardiac surgery with CPB (19). MP plasma concentrations after a dose of 30 mg/kg ranged from 1,000 to 7,500 ng/ml. In this model, clearance was calculated as 3.88 l/h and volume of distribution of the central compartment as 8.9 l (19). The PK values are thus very similar to the exploratory population PK analysis performed by Grosek et al. (17). The MP half-life in the study of Bocsi et al. (18) is considerably longer than those reported by Grosek et al. (17) and Hornik et al. (19). This may have been caused by the way clearance was calculated.

### Dose

There is high variability in the administered dose of MP (1, 32).

Centers in the US use either 30 mg/kg (9.5%) or 1 mg/kg (46%) (1); in the UK, the dose of MP used is higher (15–30 mg/kg in 50% of centers) (31).

Several studies have been performed to determine the effect of the dose of MP on inflammatory mediators and clinical outcomes. In a randomized double-blind study of 30 children 1–18 months old undergoing total TOF repair, MP 30 mg/kg was compared to MP 5 mg/kg at the induction of anesthesia. There were no differences in interleukin (IL)-6,−8, or−10 concentrations and leukocyte count (16). There were also no significant differences in TnT concentrations, SvO2, lactate concentrations, inotropic scores, or levels of NT-proBNP, suggesting no effects of dose on the cardioprotective effects of MP (16).

In a randomized study of 30 children with a mean age of 4 years undergoing repair of various congenital heart defects with CPB comparing MP 30 vs. MP 2 mg/kg before the onset of CPB performed 15 years prior, there was also no superiority of a higher dose of MP on IL-6 and−8 concentrations, C-reactive protein, and neutrophils (20). There were also no differences in 48-h clinical outcome parameters such as parameters of oxygenation, DMV, and LOS in the ICU.

Simulations of the effect of MP in a dose of 10 mg/kg and a dose of 30 mg/kg on IL-6 and IL-10 concentration–time profiles based on a newly developed PK/pharmacodynamic model in neonates undergoing cardiac surgery with CPB showed no differences in the area under the curve for IL-10 (19), for the IL-6 area under the curve was significantly but minimally lower for a 30-mg/kg MP dose compared to a 10-mg/kg dose (*p* < 0.01). The area under the curve for the IL-6 concentration–time profiles was also significantly but minimally lower following addition of a preoperative dose (*p* < 0.01) (19). Similar results were obtained after stratification for postmenstrual age, RACHS-1 risk category, and CPB time (19). The authors thus conclude that a single dose of MP of 10 mg/kg administered in the CPB prime fluid is sufficient to achieve adequate suppression of IL-6 concentrations and enhancement of IL-10 concentrations in their study population (19).

It is intriguing that there is no difference in IL concentrations between high- and low-dose MP. It may be concluded from these studies that there is no significant dose–response effect in *in vivo* studies.

### Timing

There is also much debate on the appropriate timing for corticosteroid administration in pediatric cardiac surgery. In several surveys, most practitioners give MP at induction of anesthesia or in the prime fluid of the CPB system (1, 31, 32). In a randomized study comparing MP 30 mg/kg in the pump prime to MP 30 mg/kg at anesthesia induction in 45 children between 1 and 18 months of age undergoing VSD or AVSD repair, there was no difference in mean MP concentrations between the different treatment groups, even though the peak MP concentration was achieved 52 min earlier in the induction group (15). There were no significant differences in IL-6 and IL-10 concentrations, but IL-8 concentrations were lower after weaning from CPB and 6 h later in the induction group. There were no differences in outcome measures like TnT, inotrope score, and SvO2, but the study was not powered for outcome.

Given the genomic mechanism of action of corticosteroids, early administration has been advocated. In a randomized study in 68 neonates comparing MP 30 mg/kg as a single dose intraoperatively to a two-dose regimen including an intraoperative and a preoperative dose 8 h before surgery, no superiority was shown of additional preoperative administration of MP on postoperative pro- or anti-inflammatory cytokines in neonates, even though preoperative cytokines were lower in the two-dose group (22). There were also no significant effects on outcome measures, such as LCOS, LOS in the ICU, and LOS in the hospital, but the study was not powered for outcome measures. In another randomized study in 54 children younger than 2 years old comparing MP 30 mg/kg on the evening before surgery to administration at anesthesia induction or in the prime fluid of the CPB system, there was no decreased inflammatory response in patients who received MP on the preoperative day (23). No difference in effect was shown on clinical outcome parameters such as DMV, diuresis, fluid balance, weight gain, and LOS in the ICU. The pump prime group showed a trend toward less inotropic support, shorter LOS, and less oxidative stress, but the study was, again, not powered for outcome measures. In a randomized study of 50 children younger than 5 years, MP 30 mg/kg at the beginning of surgery was compared to a two-dose regimen including a 30-mg/kg dose 4 h before surgery (9). Again, no difference in effect was shown on the concentrations of pro- and anti-inflammatory mediators. A large database study in neonates showed no significant benefit of any MP regimen (preoperative day only, pre- and operative day, operative day only) on in-hospital mortality, LOS ICU, LOS in hospital, and infection (12).

It is not known whether the results from these studies indicate that corticosteroids work mainly through their non-genomic effects or that the genomic effects of corticosteroids take place faster than assumed.

### Side Effects

There are several side effects of corticosteroid treatment which may be worrying.

Several randomized studies have shown that MP is associated with a higher incidence of hyperglycemia which needs treatment with insulin (13–16). This effect has been shown for doses ranging from 30 to 2 mg/kg (17, 20 18). In a study comparing MP 30 vs. 5 mg/kg, a significant difference in glucose levels was found, with higher glucose levels 6 h after CPB and the first postoperative day in the group receiving MP 30 mg/kg 6 (20). It cannot be concluded that lower MP doses lead to lower glucose concentrations though, given the fact that similar glucose concentrations have been reported after an MP dose of 30 mg/kg and a dose of 2 mg/kg by the same group of investigators (17, 18). The significance of hyperglycemia in pediatric cardiac surgery is still under debate. In a prospective cohort study of 379 children with a mean age of 52 months (range 0.2–180 months), undergoing repair or palliation of a congenital heart defect and receiving MP 30 mg/kg showed an incidence of hyperglycemia (glucose concentration >7 mmol/l) of 86 % (35). Severe hyperglycemia, defined as glucose concentration >11.1 mmol/l, was associated with higher mortality and more infections in a multivariate analysis (35). In a retrospective study of 144 infants weighing <10 kg undergoing cardiac surgery with CPB, no effect of hyperglycemia on outcome was seen in a multivariate analysis (36). In a multicenter randomized controlled trial comparing tight glucose regulation to standard care in the ICU in 980 patients aged 0–36 months undergoing pediatric cardiac surgery with CPB, there was no influence of tight glucose regulation on the incidence of infection, mortality, LOS in the ICU or in the hospital, and organ-specific endpoints (37).

In a large database study in 3,180 neonates receiving any regimen of MP, a multivariable analysis showed a significant association of MP with infection (OR 2.6, 95% confidence interval 1.3–5.2) in lower-risk surgical patients (STS-European Association of Cardio-Thoracic Surgery (EACTS) categories 1–3) (12). Hospital-acquired infections (central line-associated sepsis, ventilator-associated pneumonia, catheter-associated urinary tract infections, and surgical wound infections) after pediatric cardiac surgery are associated with increased LOS in the hospital, increased cost, and increased mortality (OR 2.5, 95% confidence interval 1.9–3.4) in univariate analysis in a large database study of 46.169 admissions (10). An increased incidence of infection with corticosteroid administration is thus an important signal.

It has been suggested that high-dose MP does not translate to better outcomes due to suppression of the hypothalamic–pituitary–adrenal (HPA) axis. Indeed, adrenal insufficiency has been shown in patients receiving higher doses of MP. In a prospective observational study of 119 children older than 3 months undergoing cardiac surgery with CPB receiving 30 mg/kg of MP before initiation of CPB, 60.5% of children met the criteria for adrenal insufficiency 18 h after surgery (24). None of these patients had a higher inotrope requirement, and lactate concentrations were not increased. In a retrospective study of 40 neonates who received MP 10 mg/kg 8 and 1 h before surgery, the incidence of adrenal insufficiency, as defined by a change in serum cortisol concentrations <9 mcg/dl from baseline 30 min after ACTH stimulation, was 32.5%(24). This was associated with hemodynamic compromise, with higher lactate concentrations and greater need for fluid resuscitation. In a randomized study of 40 neonates, MP 2 mg/kg was followed by a tapering dose of hydrocortisone over the first five postoperative days or until discharge from the ICU was compared to placebo (14). Postoperative cortisol concentrations were lower than 5 mcg/dl in 37 of 40 neonates (which defined hypocortisolism in this study), with no significant differences between treatment groups. There was no significant difference in the number of patients with a subnormal response to an ACTH stimulation test. It was concluded that suppression of the HPA axis, as defined by low baseline cortisol and a subnormal response to ACTH stimulation, does not take place after a dose of 2 mg/kg followed by a hydrocortisone infusion. The possibility thus exists that high-dose corticosteroid administration leads to adrenal insufficiency with hemodynamic consequences. If postoperative stress dose corticosteroids are not administered after high-dose MP, this may lead to suppression of the stress response.

### Outcome

Unfortunately, most randomized studies are too small to make definitive conclusions about outcome with MP use. One exception is a randomized study comparing MP 30 mg/kg or placebo after the induction of anesthesia in 174 neonates having surgery in two US centers (11). The study's primary outcome was powered to a previously validated morbidity–mortality composite including events following surgery before discharge including death, mechanical circulatory support, cardiac arrest, hepatic injury, AKI, or lactate > 5mmol/l. There were no reduced odds of the primary outcome after administration of MP. There were no differences in the individual components of the primary outcome and secondary outcomes of fluid balance, AKI, LCOS, DMV, LOS in the ICU, LOS in the hospital, glucose concentrations, insulin use, or infection (11). There was a decrease in the postoperative inotrope score up to 36 h after surgery. Subgroup analysis showed a significant interaction between treatment effect and treating center: in the center contributing the largest number of patients, MP was protective for the primary composite endpoint in palliative surgeries and overall, and for LCOS (11). It thus appears that small differences in patient demographics, surgical techniques, and perfusion management may still have an effect on outcome that is potentially greater than the effect of corticosteroid use.

Meta-analyses have been used as a means of overcoming the problems associated with small randomized studies. A recent Cochrane review of eight randomized studies was performed addressing the use of corticosteroids in pediatric cardiac surgery (25). There were no differences in in-hospital mortality, all-cause mortality at longest follow-up, cardiovascular mortality at longest follow-up, or LOS in the ICU. DMV was improved (25). The authors state that they cannot provide implications for clinical practice and highlight that adequately performed and well-conducted randomized trials are necessary, powered for outcome variables, and not inflammatory mediators (25). Another recently performed meta-analysis included 17 studies (26). The authors found no differences in mortality, DMV, incidence of LCOS, infection, AKI, and LOS in the ICU. Corticosteroid therapy was associated with increased blood glucose concentrations on postoperative day 1 and a higher risk of postoperative use of insulin. The inotrope score on postoperative day 1 was reduced (26).

A number of large database studies have been performed. In a database study including 46,730 patients from 38 US centers with a median age of 8 months (10), 25,113 of these patients received corticosteroids; 69.8% received MP. In a multivariate analysis, there was no difference in mortality or DMV. Patients who received corticosteroids had longer LOS in the ICU, had more infections, and had more insulin use. There was no significant benefit of corticosteroids when stratified for RACHS-1 risk category. On the contrary, in the lower-risk categories, corticosteroids were associated with increased morbidities and in RACHS-1 risk category 1 corticosteroids were associated with a higher mortality (10).

The same research group performed another database study focusing on 3,180 neonates from 25 different US centers receiving MP on the day before (12%), the day of (28%), or both on the day before and the day of surgery (22%) (12). Thirty-eight percent of patients did not receive MP. There were no differences in the primary outcomes of in-hospital mortality or LOS in the hospital. There were also no differences in the secondary outcomes of LOS in the ICU and infection. In the low-risk patient groups, use of MP was associated with an increased risk of infection across all MP regimens (OR 2.6, 95% confidence interval 1.3–5.2) (12).

Another study was performed in 549 neonates from 15 US centers undergoing a Norwood procedure who were included in the Pediatric Heart Network's Single Ventricle Reconstruction trial (27, 38). A percentage of 90.7% of these patients received intraoperative corticosteroids. In multivariate analysis, there was a trend toward increased hospital mortality in the group of patients receiving corticosteroids (OR 3.52, 95% confidence interval 0.98–12.64, p 0.054) (27). There was no difference in DMV, cardiopulmonary resuscitation, use of ECMO, infection, AKI, LOS in the ICU, LOS in the hospital, and neurodevelopmental outcomes (27).

As has been described in a structured search of the literature, there is a lack of long-term follow-up of the effect of corticosteroid treatment on late neurocognitive outcomes (39). These authors highlight that treatment with corticosteroids may lead to adverse neuromotor and cognitive function outcomes at the school age (39).

### Conclusion

We can conclude that there is real evidence that perioperative use of MP leads to a decrease in pro-inflammatory mediators and an increase in anti-inflammatory mediators. However, the suggested beneficial effect on inflammatory mediators is not associated with improved outcomes in all subgroups. The inflammatory response is characterized by multiple facets, redundancy, positive feedback loops, and amplifying cascades. This may be the reason single-agent strategies fail to produce a clinically effective decrease in the inflammatory response (2). Moreover, randomized controlled studies with enough power are just starting to be published (11).

Aside from this, there is sparse information concerning the PK and pharmacodynamics of corticosteroids. There are few studies reporting MP concentrations achieved at certain doses. It is also unknown whether changes in plasma inflammatory mediators are the appropriate pharmacodynamic target.

The lack of evidence pertaining efficacy and safety concerning corticosteroids in pediatric cardiac surgery leads to large differences in practice internationally. Further research is thus necessary, which should focus on the usefulness of corticosteroids in improving the outcome of pediatric cardiac patients, indications for corticosteroid use, and dose and timing of administration. It is unknown whether the side effects of corticosteroids outnumber its benefits. Also, there is no information on the long-term outcome of children receiving corticosteroids in high doses during pediatric cardiac surgery.

Even though the logic behind the use of corticosteroids seems sound and has led to corticosteroids being used frequently in pediatric cardiac surgery, the inconsistent clinical benefit and lack of knowledge of short- and long-term adverse events makes this practice questionable. So why do doctors use treatments that are not based on sound evidence? There are multiple reasons: our expectation for the benefits of treatment may be too high, we are looking at the wrong outcome, or just finding it difficult to abandon practices we have learned during training (40). We cannot help but ask ourselves: who are we treating? Our patients, or ourselves?

A randomized placebo-controlled double-blind study is currently underway, with an estimated completion date of July 28, 2021 (28). The STRESS trial (https://clinicaltrials.gov/ct2/show/NCT03229538) will enroll 1,200 children under the age of 1 year, with a targeted minimum of neonates <30 days, undergoing cardiac surgery with CPB. The safety and efficacy of a single dose of MP 30 mg/kg in the pump prime will be compared to placebo. This study is long overdue, and we are eagerly awaiting its results.

A fascinating new way to help us along in further research concerning the topic of the inflammatory response to cardiac surgery and CPB and the modulating effect of corticosteroids on this response is the integration of PK and pharmacodynamic modeling with systems biology in an approach called quantitative systems pharmacology (QSP). A system of coupled and interacting biological components has properties that add up to more than the sum of its separate parts. QSP describes the known components of a biologic system and integrates this information with PK and pharmacodynamics models to understand more fully the complex interaction between drug and disease biology (41). In this way, we can further build on the “three pillars of drug action”: achieving the desired drug exposure at the appropriate site of clinical action, optimal interaction of the drug with the target receptor over time, and understanding the post-receptor signaling that gives rise to the efficacious or toxic response (41). QSP may thus describe the inflammatory response to surgery, the additive effect of CPB to this inflammatory response, and how and where we can influence this process with pharmacological means.

## Author Contributions

AS performed literature research and wrote the manuscript. All authors contributed to manuscript revision and approved the submitted version.

## Conflict of Interest

The authors declare that the research was conducted in the absence of any commercial or financial relationships that could be construed as a potential conflict of interest.

## Publisher's Note

All claims expressed in this article are solely those of the authors and do not necessarily represent those of their affiliated organizations, or those of the publisher, the editors and the reviewers. Any product that may be evaluated in this article, or claim that may be made by its manufacturer, is not guaranteed or endorsed by the publisher.
